# Smoking-induced control of miR-133a-3p alters the expression of EGFR and HuR in HPV-infected oropharyngeal cancer

**DOI:** 10.1371/journal.pone.0205077

**Published:** 2018-10-05

**Authors:** Reniqua House, Mrinmoyee Majumder, Harinarayan Janakiraman, Besim Ogretmen, Masanari Kato, Evren Erkul, Elizabeth Hill, Carl Atkinson, Jeremy Barth, Terrence A. Day, Viswanathan Palanisamy

**Affiliations:** 1 Department of Biochemistry and Molecular Biology, College of Medicine, Medical University of South Carolina, Charleston, SC, United States of America; 2 Stony Brook University School of Medicine, Stony Brook, NY, United States of America; 3 Department of Otolaryngology and Head and Neck Surgery, College of Medicine, Medical University of South Carolina, Charleston, SC, United States of America; 4 Department of Otorhinolaryngology, Gulhane Medical School, University of Health Sciences, Istanbul, Turkey; 5 Department of Public Health Sciences, College of Medicine, Medical University of South Carolina, Charleston, United States of America; 6 Department of Microbiology & Immunology, College of Medicine, Medical University of South Carolina, Charleston, SC, United States of America; 7 Department of Regenerative Medicine & Cell Biology, College of Medicine, Medical University of South Carolina, Charleston, SC, United States of America; Saint Louis University, UNITED STATES

## Abstract

**Purpose:**

Human papillomavirus (HPV) infected oropharyngeal squamous cell carcinoma (OPSCC) patients have a better prognosis compared to HPV(-) counterparts. However, a subset of HPV(+) patients with a smoking history fail to respond to the standard of care treatments such as radiation and chemotherapy. To understand the underlying mechanism driving HPV(+) OPSCC patient resistance to treatment and recurrence, we sought to identify and characterize the differentially expressed miRNAs and their target genes in HPV(+) smokers and non-smokers.

**Experimental design:**

MicroRNA expression analysis was performed using Nanostring in tumor tissues isolated from a prospective cohort of HPV(+) smoking (n = 9) and HPV(+) (n = 13) non-smoking OPSCC patients. Identified miRNAs of interest were further validated using qRT-PCR in cigarette smoke extract (CSE) treated HPV(+) and E6/E7 overexpressing HPV(-) cells.

**Results:**

In comparison to OPSCC HPV(+) non-smokers, 38 miRNAs were significantly altered in the HPV(+) smoker patients cohort and out of that 9 were downregulated. Altered miRNA expression was also detected in the serum and metastatic lymph nodes of HPV(+) smokers versus non-smokers. Expression of miR-133a-3p was significantly downregulated in OPSCC smokers, HPV(+) cells and E6/E7 overexpressing HPV(-) cells treated with CSE. Reduction of miR-133a-3p induced the upregulation of miR-133a-3p target mRNAs EGFR and HuR.

**Conclusions:**

Our results indicate that miR-133a-3p is a target of smoking-induced changes in HPV(+) patients and alters the expression of EGFR and HuR which may promote HPV associated oropharyngeal cancer. Therefore, future treatment strategies for HPV(+) OPSCC smokers should focus on EGFR inhibition and the development of selective therapies to target HuR.

## Introduction

Head and neck squamous cell carcinoma (HNSCC) remains a significant threat worldwide and with five-year survival rates ranging from 20 to 75% depending on the etiopathogenesis, stage and local to distant cancer spread. Most of the HNSCC cases reported to date are the primary effects of the excessive use of carcinogens such as tobacco or alcohol. However, the emergence of high-risk human papillomavirus (HPV) infection increases the incidence of HNSCC, too [1]. HPV infection mostly occurs in the oropharyngeal (base of tongue and tonsil) squamous cell carcinoma (OPSCC) subsites of HNSCC. Interestingly, the annual number of HPV(+) OPSCC cases is expected to surpass that of HPV(+) cervical cancer cases by 2020 [2]. HPV(+) OPSCC is more likely to occur in non-smokers, tends to carry a relatively better prognosis and harbors different gene expression patterns than non-HPV tumors [3]. In fact, it was estimated that HPV(+) patients have a 60% reduction in the risk of mortality in comparison to HPV(-) patients [4]. In the United States, the incidence of HPV(+) oropharyngeal cancers is higher in men and Caucasians than in women and other races [5]. The survival of HPV(+) oropharyngeal cancer patients is reduced by alcohol consumption and smoking [6] and these patients with altered expression of EGFR, p16, p53, and Bcl-xL are associated with a reduced prognosis [7]. However, the molecular mechanism behind the poor survival rate of HPV(+) patients with a past or current smoking history still remains unknown.

Post-transcriptional gene regulation (PTR) is controlled by miRNAs and RNA-binding proteins (RBPs). PTR is causally associated with cancer progression through controlling gene expression. The changes in miRNA levels can alter PTR and correlate with local and distant metastasis in a variety of tumors [8]. MicroRNAs are known to serve as biomarkers for cancer and their expression in HNSCC has been extensively studied for their role in the clinical behavior of HPV(-) oral tumors. For example, there was a clear distinction between miR-127-3p and miR-363 expression patterns in HPV(+) and HPV(-) tumors [9]. Also, miRNAs are reported to be a predictor of smoking-related changes in human bronchial airway epithelium [10]. Although miRNA expression changes have been tested in HPV(+) and HPV(-) oral tumors [9, 11, 12], neither the expression of miRNA in HPV(+) smokers nor the effect of smoke-induced miRNA expression has been validated. Furthermore, it is unclear what specific role the smoking-mediated miRNAs play in controlling their specific targets in the HPV infected OPSCC patients.

In this study, we assessed the miRNA expression landscape in HPV(+) smokers and non-smokers in OPSCC tissues. Surprisingly, the regulation of miR-133a-3p by smoking enhanced the expression of the epidermal growth factor receptor (EGFR) and RBP Hu-antigen R (HuR). Furthermore, this mode of regulation also correlated well with smoke-induced HPV(-) cells overexpressing E6/E7 genes in trans. Both HuR and EGFR are often dysregulated in OPSCC and EGFR is a commonly targeted pathway in human oral cancer. Hence, this study directly links the smoking-induced miRNAs regulating the expression of HuR as well as EGFR that is considered as one of the prime targets for oral cancer therapy.

## Materials and methods

### Study population and sample collection

A prospective multidisciplinary study was designed to accrue patients with OPSCC at Hollings Cancer Center at MUSC. We recruited HPV(+) patients who underwent OPSCC treatment at Medical University of South Carolina through the Head and Neck Tumor Center. This study was approved by the Medical University of South Carolina Institutional Review Board. All the participants had been informed and obtained written consent for the participation of the study. Patients were eligible if they had previously untreated, advanced-stage (III, IVa-b), pathologically confirmed OPSCC, and were HPV positive. For participants, detailed smoking history was obtained, including cumulative tobacco exposure (measured in pack-years), the age when they began smoking, and second-hand tobacco exposure. Subjects with significant second-hand environmental cigarette exposure, respiratory symptoms, or regular use of inhaled medications were excluded. OPSCC HPV(+) patient biopsies were obtained during routine visits to the Head and Neck Clinic at Medical University of South Carolina. The biopsies were immediately placed in RNAlater cell reagent (Qiagen) and stored at 4°C until RNA was isolated by using the miRNeasy mini kit (Qiagen). The biopsy tissue slides and reports from the studied cases were reviewed, and the representative tumor block was selected from each patient to perform p16 immunohistochemistry (IHC) and PCR. The p16 IHC positive tissues were further selected for testing of HPV16 and 18 by PCR.

### NanoString miRNA expression analysis

Total RNA was extracted from human tissues and was subjected to Nanostring nCounter miRNA sample preparation according to the manufacturer’s instructions (Nanostring Technologies, Seattle, WA, USA). The nCounter Human miRNA Panel v2 was used to evaluate the expression of 770 miRNAs. NanoString nCounter data were analyzed using the R Bioconductor library NanoStringDiff [13], an approach which facilitates analysis of nCounter data using a negative binomial generalized linear model (GLM) with differential expression (DE) analysis performed using model-based linear contrasts, and associated inference based on likelihood ratio tests. The negative binomial GLM incorporates normalization factors for positive controls, background noise, and housekeeping genes. For evaluation of DE between smokers and non-smokers, we controlled the false discovery rate at 5%.

### Quantitative Real-Time PCR analysis

qRT-PCR was used to confirm the selected miRNAs and target mRNAs. For serum miRNA levels, *C*. *elegans* cel-miR-39 was used as a spike-in control during the miRNA conversion to cDNA using the TaqMan Advanced miRNA Assay kit (Applied Biosystems). For miRNAs, 10ng of total RNA was used in a Taq-Man Advanced assay (Applied Biosystems) as per the manufacturer’s protocol. Next, to confirm the differential expression of miR-154-5p, miR-551b-3p, miR-652-3p, miR-133a-3p and let-7d-5p, the data was normalized to the expression of cel-miR39 for the spike in unless otherwise mentioned. For mRNA, 1 μg of starting total RNA was used for qRT-PCR of miR-133a-3p targets including EGFR, HuR, ABCC2, CASP3, MCL1, RHOA, and XIAP and were all normalized to expression of GAPDH, Actin, and RPS18 (S1 Table) using the SYBR green protocol (Applied Biosystems). For both miRNA and mRNA quantitation from tissues and cells, each PCR was run in triplicate. After amplification of 40 cycles, the data acquisition was carried out with StepOnePlus Real-Time PCR system (Applied Biosystems). Data from all qRT-PCR experiments were analyzed using the comparative Ct method, and replicates were filtered by using a *q-*test, with outliers being removed at 90% confidence.

### Prediction of miR-133a-3p mRNA targets

Putative mRNA targets for miR-133a-3p (inferred targets derived from seed) and pathways mapping to the mRNAs were obtained using Targetscan and Ingenuity Pathway Analysis software (Qiagen), respectively. Biological process enrichment analysis was conducted on mRNA targets using ToppFun (toppgene.cchmc.org/enrichment.jsp) [14]. Interactions between the 89 targets belonging to the significantly enriched category cell migration (GO:0016477;p = 1.8e^-5^) were mapped using String analyses (https://string-db.org/cgi/input.pl) [15].

### Cell lines, antibodies and plasmids

HNSCC cell line HPV(-) UMSCC11A, obtained from University of Michigan, HPV(+) cells UMSCC47 and HPV(-) SCC1A were gifts from Ogretmen laboratory, Biochemistry and Molecular Biology, MUSC, Charleston, SC, USA. Cell lines were routinely checked for mycoplasma contamination. SCC1A was grown in Dulbecco’s modified Eagle medium (DMEM-Hyclone) containing 10% fetal bovine serum (FBS) (Atlanta Biologicals), with 100U/ml penicillin and 100 μg/ml streptomycin. UMSCC11A was grown in Dulbecco’s modified Eagle medium (DMEM-Hyclone) containing 10% fetal bovine serum (FBS) with 100U/ml penicillin and 100 μg/ml streptomycin and 1% non-essential amino acids. UMSCC47 was grown in DMEM with 10% FBS, 2 mM l‐glutamine, 1× non‐essential amino acids solution, and 500 μg/ml gentamicin (Gibco). HuR antibody was obtained from Santa Cruz (cat# sc-5261), EGFR antibody obtained from Cell signaling technology (Cat# 2232S), β-Actin monoclonal antibody was purchased from Proteintech (cat # 60008-1-IG). HPV E6/E7 expressing plasmid p1321 HPV(-)16 E6/E7 was a gift from Peter Howley [16] (Addgene, catalog # 8641).

### Cigarette smoke exposure in cells

Cigarette smoke extract was generated as described [17] by bubbling smoke from four 3R4F cigarettes (University of Kentucky) through 50 ml of media using a Shapiro cigarette smoke machine (Washington University, St. Louis, MO). Media was filter-sterilized (0.22 μm) and used immediately. CSE was diluted with fresh media to create 5, 10, and 20% final concentrations. The cells were treated with increasing percentage of CSE, and untreated cells were serving as controls. The potential toxicity of this concentration of CSE was evaluated in cell growth assay by cyQuant (Thermo Fisher). Cells were harvested at different times of post-exposure, and RNA was isolated from these cells by using the miRNeasy mini kit according to manufacturer’s protocol.

### Statistical analysis

Few data points are expressed as the mean ± the standard deviation. Two-sample t-tests with equal variances are used to assess differences between means. Results with *p* values less than 0.05 are considered significant.

## Results

### Smoking alters the expression of a subset of miRNAs in HPV(+) OPSCC tissues

**Fig 1:**
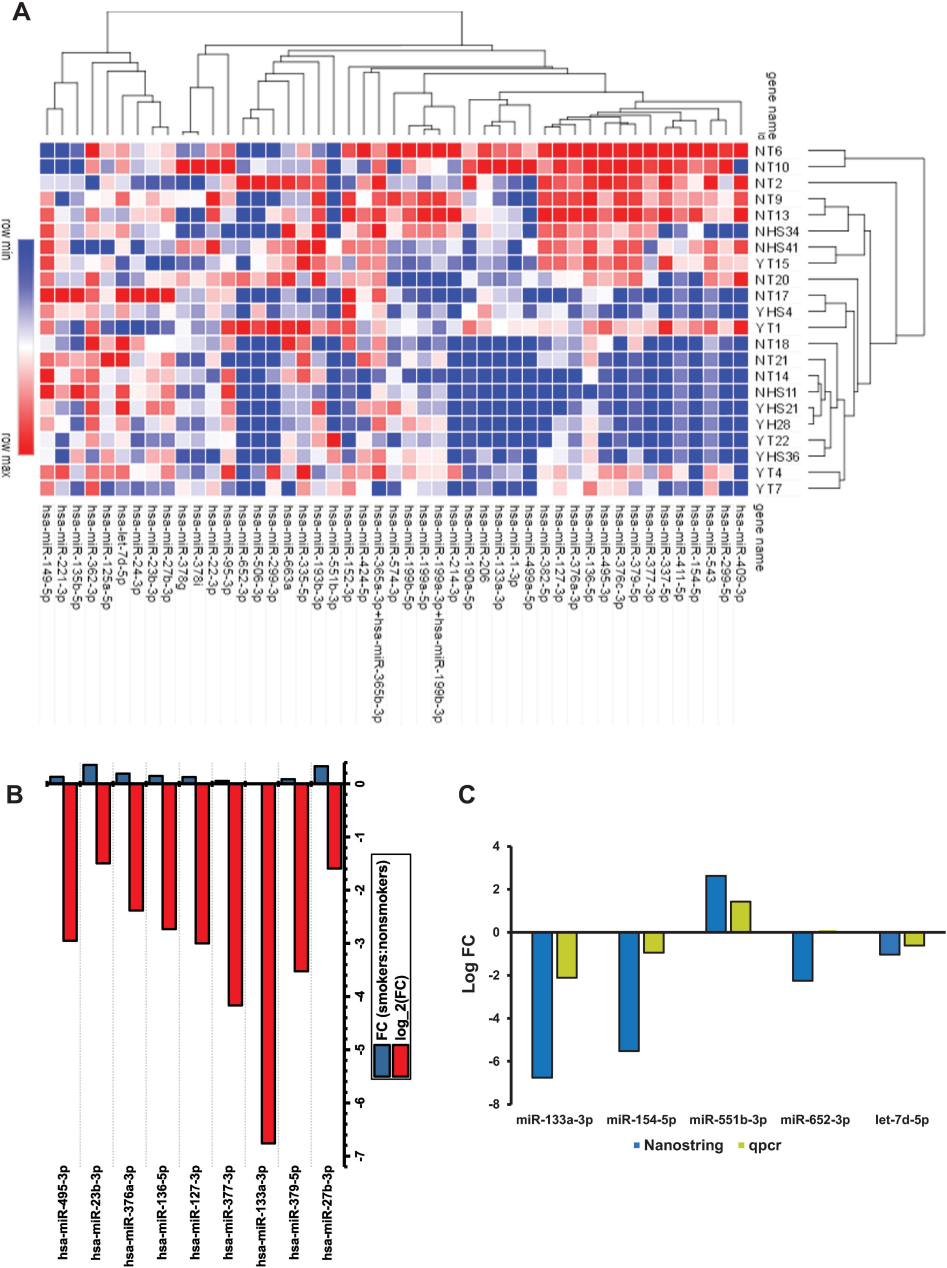
Altered expression of miRNAs in HPV(+) smokers OPSCC. (**A**) A heat map of the normalized read counts of the miRNAs that are identified as differentially expressed between smokers and non-smokers of HPV(+) OPSCC tissues. Hierarchical clustering revealed the separation of the smokers and non-smokers is based on miRNA expression data (more red color correlates with the more highly expressed miRNAs). (**B**) Top 9 miRNAs were downregulated in OPSCC patient samples. Bar chart showing the mean logarithmic fold change (RQ) of the top nine downregulated miRNAs in smoker samples relative to non-smokers. (**C**) Comparison of Nanostring and qRT-PCR miRNA expression levels in OPSCC tissues. Nanostring assay selected up- and down-regulated miRNAs were cross-validated with the qRT-PCR chart of the logarithmic fold change of the top five altered miRNAs.

MicroRNA expression in OPSCC tissues from the 22 HPV(+) patients admitted to Medical University of South Carolina was determined by using the Nanostring miRNA expression assay. The patient cohort consists of 9 current or past smokers and 13 never smokers, and the study design is illustrated in supporting information (S1 Fig). Table 1 depicts the demographic data of subjects recruited for the study. The HPV status of the biopsies was determined by the expression of p16^INK4a^ immunohistochemistry in the clinical pathology laboratory. The p16 IHC indeed is a recommended prognostic test in the clinical practice as an alternative for PCR. Also, p16 immunohistochemistry can be performed at low cost using routine laboratory techniques. Because p16 is upregulated after high-risk HPV infection, p16 IHC can be used as an alternate marker for high-risk HPV integration [18–20]. To further confirm the collected biopsies or surgically resected tissues are positive for HPV infection, we carried out semi-quantitative PCR for HPV types 16/18 using gene-specific primers (S2 Fig). The tissue biopsies indicated the presence of HPV 16 or 18 were selected for Nanostring miRNA analysis. After Nanostring data processing using nSolver Analysis Software, the detected miRNAs between smokers and non-smokers were identified, and the quantitative expression of these miRNAs was chosen for further analysis. Based on the *q* value (*p*-value adjusted to the false discovery rate, FDR) of <0.10, 48 miRNAs (Fig 1A and S2 Table) were differentially expressed between the smokers and the non-smokers of the HPV patients. However, a *q* value of <0.05 yielded 38 miRNAs and <0.01 yielded 15 miRNAs (S2 Table) that are differentially expressed between smokers and non-smokers. Of these 15 miRNAs, 9 down-regulated miRNAs were chosen for further analysis (Fig 1B). The most significantly altered miRNA in smokers compared to non-smokers was miR-133a-3p. Five of the top differentially expressed miRNAs (miR-154-5p, miR-551b-3p, miR-652-3p, miR-133a-3p and let-7d-5p) were validated by using qRT-PCR (Fig 1C), with fold changes comparable to those determined by Nanostring analysis. Altogether, the findings indicate that a subset of miRNAs including miR-133a-3p is downregulated in HPV(+) smokers.

**Table 1:** HPV patients demographic information.

HPV+ OPSCC	Nonsmokers (N = 13)		Smokers (N = 9)	
Age, median (range), years	60 (50–69)		58 (44–70)	
Male	11 (85%)		9 (100%)	
Ethnicity				
Caucasian	12 (92%)		8 (89%)	
African American	1 (8%)		1 (11%)	
Primary Tumor Location				
Tonsil	6 (46%)		7 (78%)	
Base of the Tongue	7 (54%)		1 (11%)	
Soft Palate	0 (0%)		1 (11%)	
T-stage	Clinical	Pathologic	Clinical	Pathologic
T1	7 (54%)	7 (54%)	2 (22%)	4 (44%)
T2	4 (31%)	2 (16%)	6 (67%)	4 (44%)
T3	1 (8%)	1 (8%)	0 (0%)	0 (0%)
T4a	1 (8%)	1 (8%)	1 (11%)	0 (0%)
Tx	0 (0%)	2 (16%)	0 (0%)	1 (11%)
N-stage	Clinical	Pathologic	Clinical	Pathologic
N0	3 (23%)	3 (23%)	0 (0%)	1 (11%)
N1	1 (8%)	2 (16%)	2 (22%)	1 (11%)
N2a	2 (16%)	1 (8%)	3 (33%)	3 (33%)
N2b	7 (54%)	4 (31%)	2 (22%)	2 (22%)
N2c	0 (0%)	0 (0%)	1 (11%)	0 (0%)
N3	0 (0%)	0 (0%)	1 (11%)	1 (11%)
Nx	0 (0%)	3 (23%)	0 (0%)	0 (0%)
Stage Group	Clinical	Pathologic	Clinical	Pathologic
I	2 (16%)	2 (16%)	0 (0%)	0 (0%)
II	1 (8%)	1 (8%)	0 (0%)	1 (11%)
III	1 (8%)	2 (16%)	2 (22%)	1 (11%)
IVA	8 (62%)	5 (38%)	6 (67%)	6 (67%)
IVB	0 (0%)	0 (0%)	1 (11%)	0 (0%)
IVC	1 (8%)	0 (0%)	0 (0%)	0 (0%)
NA	0 (0%)	3 (23%)	0 (0%)	1 (11%)

### HPV(+) smokers exhibit lymph node metastasis and reduced miR-133a-3p in both lymph node and serum

**Fig 2:**
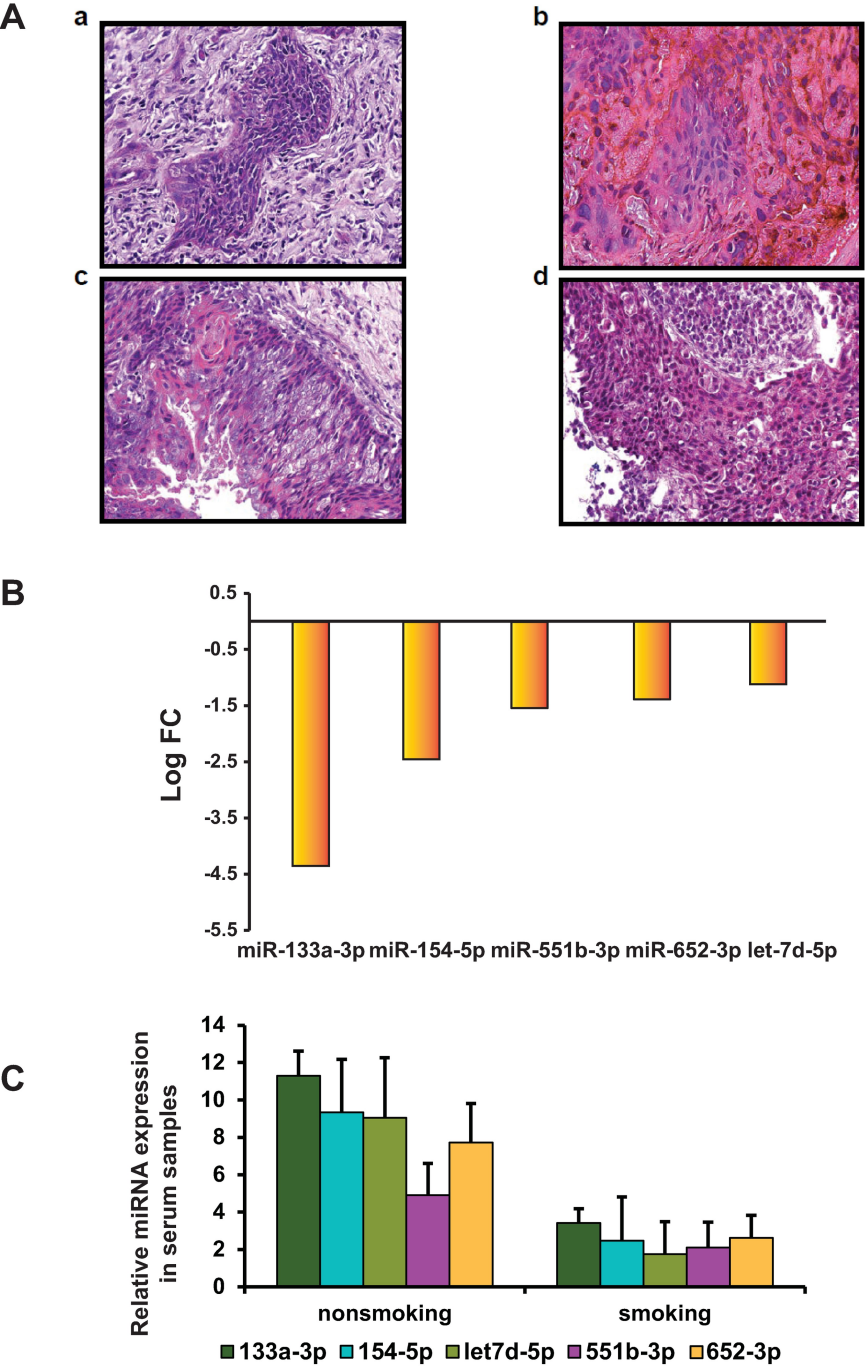
Expression of selected miRNAs in lymph node and serum of HPV(+) OPSCC. (**A**) Four lymph node smokers samples with metastatic lesions were subjected to H&E staining. Tumor islands can be seen in the fresh frozen section of lymph nodes in all four samples (H&E stain, ×100). (**B**) Bar chart of the mean logarithmic fold change (RQ) of the downregulated miRNAs in lymph node metastatic samples (n = 4) relative non-metastatic lymph nodes (n = 8). **(C)** Relative miRNA expression levels in serum samples between smokers (n = 9) vs. non-smokers (n = 13) were determine by qRT-PCR. Cel-miR-39 spike in was used to normalize the values.

The presence of nodal metastasis impacts overall prognosis and survival of head and neck cancer patients. We have obtained a total of 12 surgically removed lymph node samples from HPV(+) patients and tested for nodal metastasis. We found that within 12 HPV(+) lymph node samples (smokers n = 7, non-smokers n = 5), the presence of nodules and carcinoma in 4 samples (Fig 2A and S3 Table) indicating a possible metastatic behavior of HPV(+) smokers. To validate the miRNAs that are downregulated in these four lymph nodes, we tested the expression of miRNAs compared with the remaining eight lymph node samples that have no signs of metastasis. The qRT-PCR analysis of RNA extracted from lymph node indicates that miR-133a-3p is the most downregulated compare to other miRNAs (Fig 2B). Next, we tested the miRNA levels in the serum of these patients. To control the variability between serum samples during the extraction and measurement of the miRNAs, we *spiked*-in non-human synthetic *C*. *elegans* microRNA (cel-miR-39) after the initial denaturation of serum as described [21]. As shown in Fig 2C, all the validated miRNAs are downregulated in the smokers’ serum in comparison with the non-smokers’ serum samples. This observation indicates that miR-133a-3p is indeed downregulated in both lymph node and serum of HPV(+) patients who are either reformed or current smokers.

### Regulation of miR-133a-3p and its mRNA targets EGFR and HuR in response to in vitro cigarette smoke exposure

**Fig 3:**
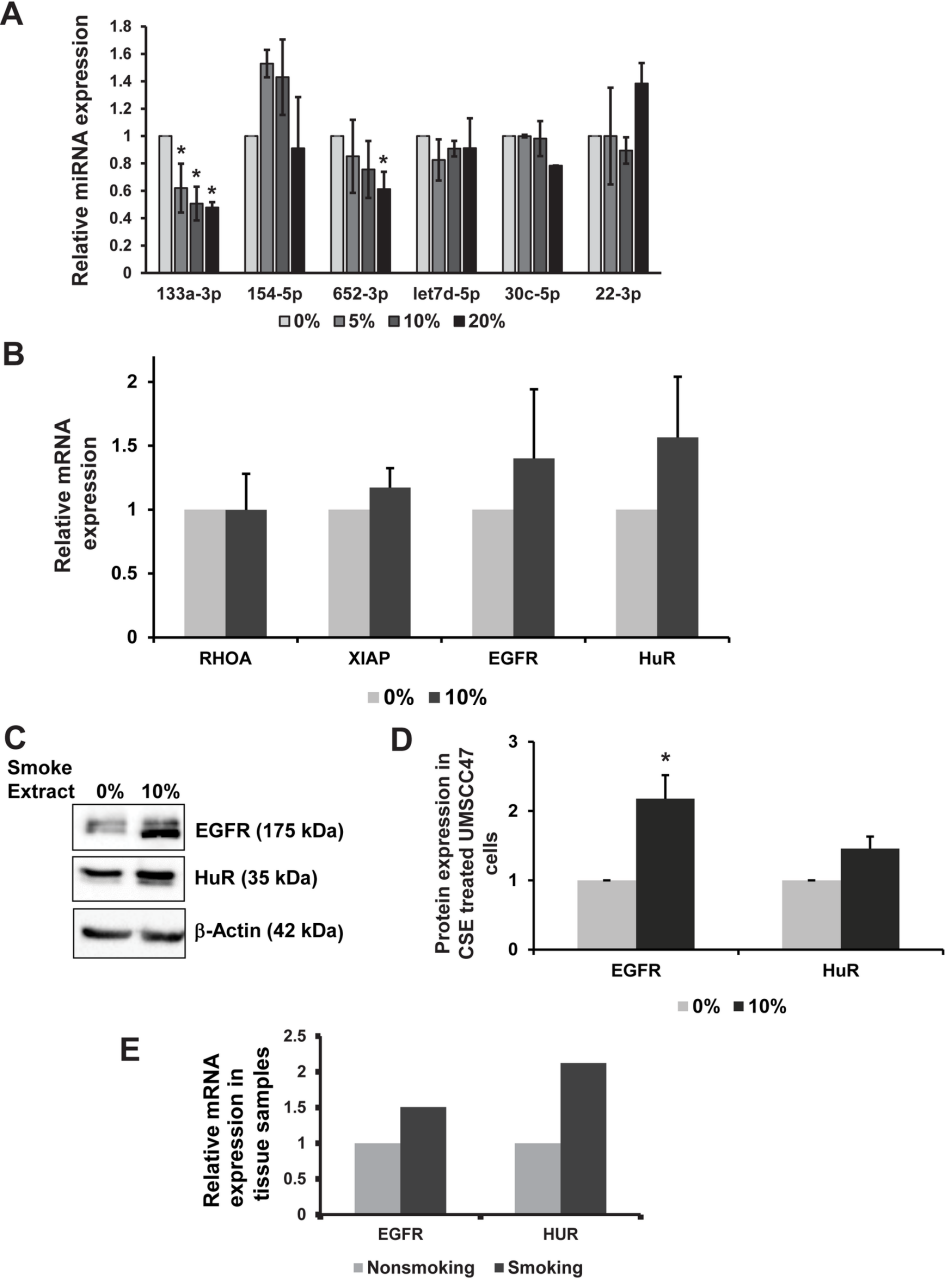
Treatment of CSE modulated miR-133a-3p in HPV(+) cells. **(A)** UMSCC47 cells were treated with different concentrations (0, 5, 10, 20%) of CSE for eight days and tested for relative expression of miRNAs. * denotes *p*-value of <0.05. **(B)** Estimation of miR-133a-3p target mRNAs. UMSCC47 cells were treated with 10% CSE for eight days and tested for indicated mRNA levels by qRT-PCR. Actin and RPS18 genes were used as endogenous controls. **(C)** Western blot analysis of EGFR and HuR in UMSCC47 cells after eight days treatment with 10% CSE. β-Actin was used as loading control. **(D)** Graphical representation of quantitative levels of HuR and EGFR protein levels. β-Actin was used for normalization. **(E)** Relative mRNA levels of EGFR and HuR in smokers (n = 9) and non-smokers (n = 13) tissue samples, as obtained by qRT-PCR. Actin and RPS18 were used as endogenous controls.

To further investigate whether smoking is responsible for the changes miRNA expression as seen in the HPV(+) smokers, we performed two critical in vitro experiments. First, we used a human HPV(+) oral cancer cell line UMSCC47 exposed to different concentrations of cigarette smoke extracts (CSE) and analyzed the expression of selected miRNAs using qRT-PCR. We found that there was a significant downregulation of miR-133a-3p in all 5, 10 and 20% smoke concentrations compared to other miRNAs (Fig 3A). This observation indicates that miR-133a-3p is repressed upon exposure to CSE and suggests that miR-133a-3p downregulation likely plays an additional role in HPV(+) tumor progression by modulating its targets. Second, to determine the functional role of miR-133a-3p in these cells, we sought to identify the mRNA targets of miR-133a-3p using a bioinformatic approach. To identify the mRNA targets of miR-133a-3p, we used multiple target prediction software including Target Scan and Ingenuity Target Explorer. We found several mRNA targets (859 mRNA targets) for miR-133a-3p and tested the top inferred miR-133a-3p targets and their biological role associated with their contribution to cancer cell growth and development. Based on the target prediction and positive regulation of gene expression, we identified two important miR-133a-3p targets such as EGFR and HuR. Next, to determine whether the repression of miR-133a-3p by smoking is required for the induction of its mRNA targets, we determined the effects of CSE exposure on the expression of miR-133a-3p mRNA targets in HPV(+) UMSCC47 cells. Upon exposure to 10%, CSE the miR-133a-3p mRNA target expression levels are increased by ~1.5-fold (Fig 3B). Out of most mRNA targets tested only two of the mRNA targets EGFR and HuR showed up-regulated protein expression under CSE (Fig 3C). The quantitative information of the protein blot is depicted in Fig 3D. Next, to determine and validate the downregulated miR-133a-3p mRNA targets EGFR and HuR expression levels in human HPV(+) tissue samples, we analyzed EGFR and HuR expression levels in extracted total RNA. As shown in Fig 3E, there is an observed increase (~2.0 fold) in the mRNA levels of EGFR and HuR. This observation suggests that downregulation of miR-133a-3p may promote the expression of its mRNA targets EGFR and HuR in the HPV(+) smokers. Altogether, these results indicate that repression of miR-133a-3p by CSE promotes the increased expression of EGFR and HuR proteins in HPV(+) smokers.

### HPV(-) oral cancer cells overexpressing E6/E7 proteins treated with CSE downregulates miR-133a-3p and upregulates EGFR and HuR

**Fig 4:**
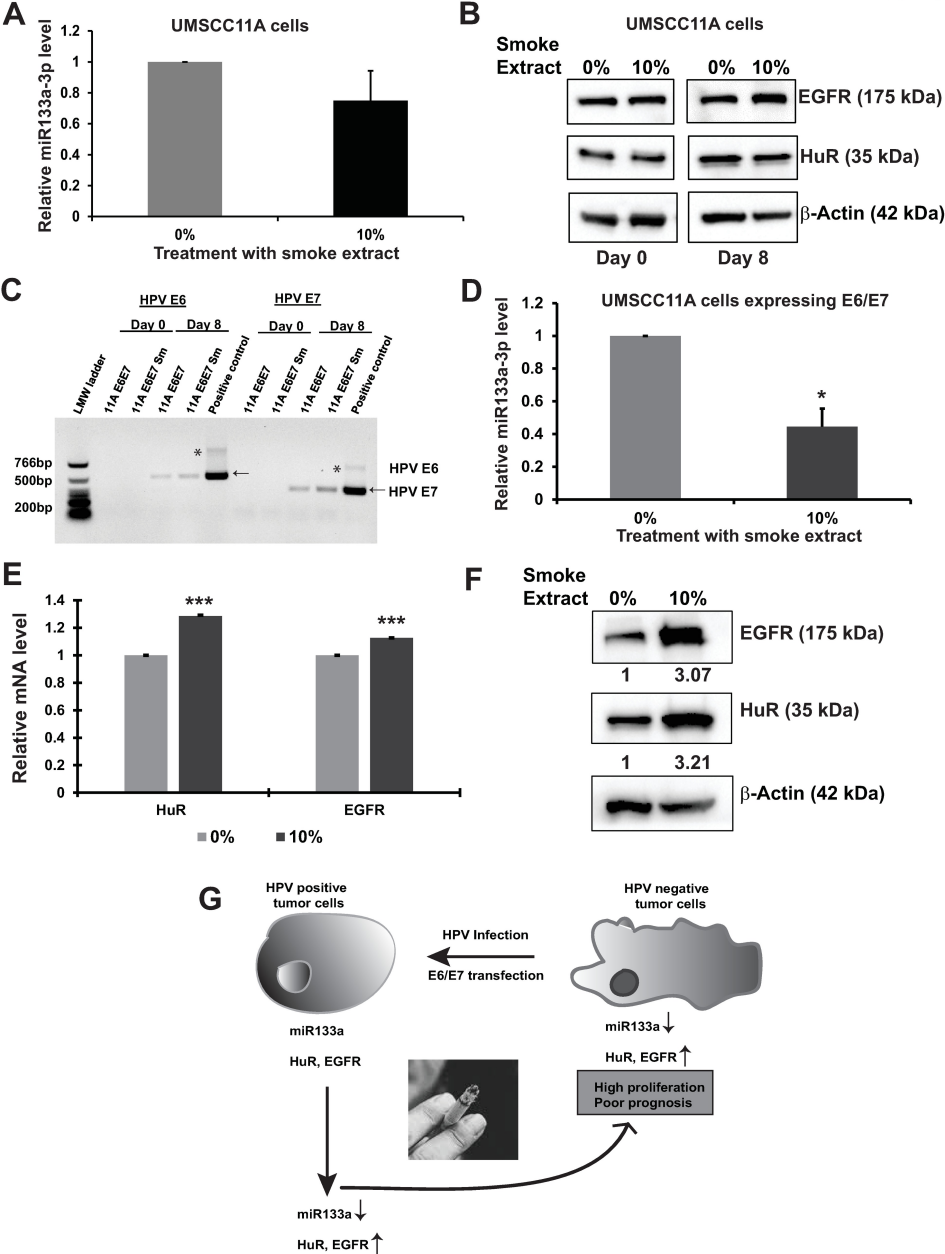
HPV(-) cells overexpressing E6/E7 plasmid downregulate miR-133a-3p expression and in turn modulate EGFR and HuR protein expression. **(A)** UMSCC11A cells were treated with 10% CSE for eight days and tested for miR-133a-3p levels by qRT-PCR. Actin and RPS18 were used as endogenous controls. **(B)** UMSCC11A cells were treated with 10% CSE for eight days and tested for EGFR and HuR proteins. β-Actin was used as loading control. **(C)** UMSCC11A cells were transfected with E6/E7 overexpression constructs, identified using semi-quantitative PCR and the DNA was separated by 2% agarose gel electrophoresis. Bands shown with arrowheads indicate the expression of E6 and E7 genes after eight days of incubation. UMSCC47 genomic DNA was used here as a positive control for E6 and E7. * denotes non-specific bands. **(D)** UMSCC11A cells overexpressing E6/E7 constructs were treated with 10% CSE for eight days, and the miR-133a-3p level was determined between zero and eight days of 10% CSE treatment. *denotes *p*-value of <0.05. U6 was used as an endogenous control. **(E)** mRNA analyses of EGFR and HuR were done after HPV infected UMSCC11A cells were treated with 10% CSE for eight days. *** denotes *p*-value of <0.0005. **(F)** Western blot analysis of EGFR and HuR levels that were tested after eight days of treatment with 10% CSE. β-Actin was used as loading control. **(G)** Our molecular model depicts HPV(+) tumor cells expressing miR-133a-3p maintain regular levels of EGFR and HuR, and smoking alters the expression of miR-133a-3p and promotes the expression of EGFR and HuR, enhancing cell proliferation thus resulting in a poor prognosis for OPSCC patients.

Next, we wanted to understand whether HPV E6/E7 proteins and the CSE are both required, simultaneously, for the downregulation of miR-133a-3p and upregulation of its targets EGFR and HuR. First, we determined the effects of CSE exposure alone on the HPV(-) oral cancer cells such as UMSCC11A and SCC1A. To test if CSE had any effect on miR-133a-3p and its targets in HPV(-) cells, we treated UMSCC11A cells with 10% CSE for eight days and looked at the miR133a-3p expression (Fig 4A). The treatment did not show any significant down-regulation in the miRNA expression unlike HPV(+) cells in Fig 3A. Next, we looked at EGFR and HuR, the targets of miR-133a-3p, protein expression in UMSCC11A cells after the same treatment with CSE (Fig 4B). Like miR133a-3p expression in Fig 4A, EGFR and HuR protein expression did not change with CSE treatment on HPV(-) cells. To see if both HPV E6/E7 and CSE were required to induce the effects on the miRNA and EGFR and HuR, we overexpressed E6/E7 in UMSCC11A (Fig 4C) and SCC1A (S3A Fig) cells and tested their expression levels after eight days of 10% CSE treatment. As shown in Fig 4C, the presence of E6/E7 mRNAs were confirmed in the eighth day CSE treated cells, by RT-PCR. Next, the RNAs isolated from these cells were subjected to qRT-PCR to test the expression of miR-133a-3p. Like HPV(+) cells in Fig 3A, the resulting data here indicated that miR-133a-3p was significantly downregulated after eight days of CSE treatment in HPV(-) cells expressing E6/E7 (Fig 4D). Next, to determine the levels of miR-133a-3p target mRNAs EGFR and HuR, the total RNA extracted from cells were subjected to qRT-PCR analysis. As expected, both EGFR and HuR mRNA levels were significantly increased (Fig 4E), and their protein levels were also upregulated more than three-fold for both EGFR and HuR (Fig 4F). Also, HPV(-) cell line SCC1A, exhibited a similar trend in EGFR and HuR mRNA and protein upregulation as observed in UMSCC11A cells (S3B and S3C Fig, respectively). Altogether, our data suggest that the modulation of miR-133a-3p levels by smoke exposure in association with HPV viral proteins contributes to the smoking-dependent regulation of EGFR and HuR expression levels in HPV(+) OPSCC (Fig 4G).

## Discussion

In clinical practice, OPSCC HPV(+) HNSCC patients respond to the conventional surgery, radiation therapy and chemoradiotherapy better than HPV(-) patients [22, 23]. However, HPV(+) patients who are current smokers or have a smoking history tend to have poor treatment outcome [6, 24]. The rationale of this study was to determine whether smoking alters the miRNA expression patterns in HPV(+) patients admitted to MUSC. We found 15 miRNAs that are differentially expressed, with most (2-fold) downregulated in smokers. Smoking is known to impact miRNA expression in airway epithelial cells [10] and other cell systems [25, 26]. In the context of HNSCC, several reports indicated that miRNAs are differentially expressed between HPV(-) and HPV(+) tumors [9, 12]. The differential expression patterns of miRNA in HPV(+) cases may be due to the combination of papillomavirus load and associated malignancy. However, the combination of HPV infection, OPSCC, and smoking severely impact the treatment outcome possibly through changes in gene expression. Interestingly, the smoking status appears to distinctly control the prognosis of HPV(+) OPSCC patients based on pack-years of tobacco smoking, tumor stage, and nodal stage [27]. This suggests that there may be an underlying mechanism which drives HPV(+) smokers poor response to treatment. Our data decoded the molecular connection between smoking and miRNAs, which plays an essential role in target gene expression and provides an opportunity to understand the complex nature of HPV(+) OPSCC.

Despite the strong relationship between miRNA and mRNA in post-transcriptional gene regulation [28], miRNAs distinctly expressed and correlated with target mRNAs in HPV(+) OPSCC have not been well documented. Although many tools are available to understand the nature of the miRNAs in cancer, there are no predictive biomarkers that distinguish the smoking-associated changes in HPV(+) patients. Our findings illustrate that miRNAs may have the potential to serve as biomarkers for HPV(+) infection associated with smoking. Furthermore, the lymph node metastasis is always a predictive prognostic marker of the disease progression and stage, but often they poorly correlate with recurrence [29]. Our data (Fig 2) indicate that the miRNAs downregulated in smoking patients’ tissues and serum exhibit the same trend as lymph nodes highlight the potential use of miRNA as biomarkers. Ultimately, detecting these miRNAs in the early stage of the HPV infection may provide a novel intervention during therapy.

Smoking alters the expression of miRNAs, in turn, their mRNA targets followed by the functions to modulate cancer progression. Estimating the levels of miRNA and target mRNAs from the same samples, we were able to identify the correlations between miRNA and mRNA expression profiles across the cohorts of smoking and non-smoking patients. For example, miR-218 and its mRNA targets in response to smoking were observed in airway epithelial cells supports the notion that smoking impact both miRNA and its target mRNA expression patterns [10]. Our data illustrated that miR-133a-3p downregulated and promoted its targets such as EGFR and HuR in HPV(+) tissues and cells. Of note, miR-133a-3p has been reported to inhibit proliferation and migration of HNSCC cells through targeting COL1A1 and repressing its expression [30].

Furthermore, miR-133a-3p has been noted as a tumor suppressor miRNA downregulated in several cancers including HNSCC tumors and also known to target EGFR [31–34]. In bladder cancer downregulated miR-133a-3p was reported to serve as a potential biomarker for bladder cancer progression [35, 36]. Also, in lung cancer patients with reduced expression of miR-133a-3p in a smoking-dependent manner implicated its role in the prognosis of current, former and never smokers [25, 26]. There are changes in gene expression between HPV(+) smokers and non-smokers, our findings suggest that the downregulation of miR-133a-3p in HPV(+) smokers promote the expression of EGFR and HuR. However, the effect of miR-133a-3p on EGFR and HuR also strongly dependent on HPV infection, suggest that HPV associated smoking habits impacts miRNA biogenesis and contribute to tumorigenesis. For example, HPV16 and HPV18 DNA load were strongly associated with smokers indicating that the possibility of HPV associated changes in smoker’s gene expression patterns [37]. Altogether, these findings demonstrate that miR-133a-3p-mediated regulation of EGFR and HuR contributes to enhanced tumor growth, and possibly promotes metastasis. Smoking is known to induce EGFR, and exhibit resistance to lung cancer treatment [38].

Interestingly, in a cohort of 42 OPSCC patients, the authors found HPV positivity in more than 64% of the tumors and identified smoking is significantly associated with higher EGFR expression, a strong predictor of outcome and response to therapy [24]. Collectively, these data suggest that smoking impacts EGFR expression levels, and our study supports the notion that mechanism of action of smoking on EGFR expression. It is not known how HPV infection reduced the expression of EGFR either mRNA or protein level. It has been hypothesized that it could be due to low *EGFR* gene copy number [39]. However few studies suggested that EGFR protein over-expression in HNSCC appears to be a post-transcriptional or post-translational phenomenon [40, 41]. However, our findings strongly support this notion that miRNAs may play a role in the altered expression of EGFR in HPV infected cells. HuR is a crucial protein that controls post-transcriptional gene regulation in multiple cancers.

Interestingly, HuR controls the expression of miR-133a-3p by a feedback mechanism, where overexpression of HuR in muscle cells binds with miR-133a-3p/b precursor long non-coding RNA linc-MD1 and control the processing of miR-133a-3p and miR-133b [42]. This observation demonstrated that overexpression of HuR might have control over the expression of miR-133a-3p. A similar interesting observation was noted in our data that CSE-dependent increase in HuR expression was observed when miR-133a-3p levels were low. These data suggest that smoking-dependent modulation of miR-133a-3p and HuR may occur possibly through feedback control measures. However CSE has an additional role in HuR expression, it is very likely that HuR-mediated control of miR-133a-3p contributes to more regulatory pathways in gene expression.

In summary, we have profiled global miRNA expression in the HPV(+) smokers and non-smokers. Our findings suggest that miRNAs may play an important role in regulating the gene expression in response to tobacco exposure in HPV(+) OPSCC patients. MicroRNA profiles obtained from patients might, therefore, serve as biomarkers for smoking-related OPSCC patients with HPV infection and help to elucidate the regulatory mechanisms that mediate patient’s response to tobacco smoke exposure (Fig 4G, model depicts the proposed study and conclusion). Based on the evidence from HPV patients and EGFR status and our data illustrate that smoking cessation strategies are needed for smokers with HPV infected OPSCC. It will be interesting to study the HPV infection associated EGFR expression and targeted use of EGFR inhibitors for those patients who are either current or past smokers. A combination of single or multiple agent treatment options could improve the outcome of those OPSCC patients. Likewise, a HuR or its associated gene network based therapeutic interventions is warranted. Results from our small sample size results provide a strong basis for the validation in a larger cohort of patients, which would improve the treatment predictions of OPSCC patients at the risk of resistance and recurrence.

## Supporting information

S1 Fig Study design: Recruitment and analysis plan of clinical specimens. (EPS)

S2 Fig Semi-quantitative PCR analysis of HPV16/18 E6/E7 genes. The PCR products of E6/E7 genes separated on 2% agarose gel and visualized under Bio-Rad gel doc. S: Samples from smokers; NS: Samples from nonSmokers.
(EPS)

S3 Fig HPV(-) SCC1A cells are expressing E6/E7 exhibit changes in miR-133a-3p, EGFR and HuR levels. **A.** UMSCC1A cells were transfected with E6/E7 overexpression constructs, identified using semi-quantitative PCR and the DNA was separated by 2% agarose gel electrophoresis. Bands shown with arrowheads indicate the expression of E6 and E7 genes after eight days of incubation. UMSCC47 genomic DNA was used here as a positive control for E6 and E7. **B.** Relative mRNA levels of EGFR and HuR after eight days of CSE treatment. **C.** Western blot analysis of EGFR and HuR levels after eight days treatment of 10% CSE. β-Actin was used as loading control.
(EPS)

S1 Table Primers used for the study are listed in this table. (DOCX)

S2 Table The miRNA expression between HPV(+) smokers vs. non-smokers, *q* values. (XLSX)

S3 Table Relationship with smoking and lymph node metastasis in the HPV(+) OPSCC. (DOCX)
