# Supplementary material for: Smoking-induced control of miR-133a-3p alters the expression of EGFR and HuR in HPV-infected oropharyngeal cancer
Source: PLoS One. 2018 Oct 5;13(10):e0205077. doi: 10.1371/journal.pone.0205077 (PMC6173415; doi:10.1371/journal.pone.0205077)
Supplement: S1 Table — (DOCX) [file pone.0205077.s004.docx]

| Primer | **Sequence** |
| --- | --- |
| EGFR-F | GCCTCCAGAGGATGTTCAATAA |
| EGFR-R | TGAGGGCAATGAGGACATAAC |
| XIAP-F | TTCCAAGTGGTAGTCCTGTTTC |
| XIAP-R | CCAACTGCTGAGTCTCCATATT |
| RhoA-F | GTGGATGGAAAGCAGGTAGAG |
| RhoA-R | TAACATCGGTATCTGGGTAGGA |
| Casp3-F | GCTGCCTGTAACTTGAGAGTAG |
| Casp3-R | GTATGGAGAAATGGGCTGTAGG |
| MCL1-F | GTGAAGATGGTAGGGTGGAAAG |
| MCL1-R | TCGGCGGGTAATCAATTCTATG |
| ABCC2-F | GAGGATGAATCTCGACCCTTTC |
| ABCC2-R | CTCTGTCACTTCGTGGGATAAC |
| GAPDH-F | GGTGGTCTCCTCTGACTTCAACA |
| GAPDH-R | GTTGCTGTAGCCAAATTCGTTGT |
| P21-F | CGGAACAAGGAGTCAGACATT |
| P21-R | AGTGCCAGGAAAGACAACTAC |
| HuR-F | GGCGTGGCTTACAGTTCCAA |
| HuR-R | CAGCGTTTAACACGAACCTGATAC |
| BetaActin-F | GGACCTGACTGACTACCTCAT |
| BetaActin-R | CGTAGCACAGCTTCTCCTTAAT |
| RPS18-F | CTTTGCCATCACTGCCATTAAG |
| RPS18-R | ATCACACGTTCCACCTCATC |
| Claudin-F | CCAGTTAGAAGAGGTAGTGTGAAT |
| Claudin-R | CAGCCAGCTGAGCAAATAAAG |
| E-cadherin-F | GTCATTGAGCCTGGCAATTTAG |
| E-cadherin-R | GTTGAGACTCCTCCATTCCTTC |
| c-Myc-F | CATAACGCGCTCTCCAAGTA |
| c-Myc-R | GGGAGGAATGATAGAGGCATAAG |
| HPV16 E6-F | CTCTGAATTCGCCACCATGCACCAAAAGAGAACTGCA |
| HPV16 E6-R | CCCTCGAGGTATCTCCATGCATGATTACA |
| HPV16 E7-F | CTCTGAATTCGCCACCATGCATGGAGATACACCTACA |
| HPV16 E7-R | CCCTCGAGGATCAGCCATGGTACATTATGG |
| HPV18 E6-F | CTATAGAGGCCAGTGCCATTCG |
| HPV18 E6-R | TTATACTTGTGTTTCTCTGCGTCG |
| HPV18 E7-F | TAATCATCAACATTTACCAGCCCG |
| HPV18 E7-R | CGTCTGCTGAGCTTTCTACTACTA |

S1 Table. Primers used for the study.
