# Supplementary material for: Smoking-induced control of miR-133a-3p alters the expression of EGFR and HuR in HPV-infected oropharyngeal cancer
Source: PLoS One. 2018 Oct 5;13(10):e0205077. doi: 10.1371/journal.pone.0205077 (PMC6173415; doi:10.1371/journal.pone.0205077)
Supplement: S3 Table — (DOCX) [file pone.0205077.s006.docx]

S3 Table. Relationship with smokers and lymph node metastasis in the HPV(+) OPSCC.

| ID | Samples | Metastasis | Nodules* | Area (%)** | Image |
| --- | --- | --- | --- | --- | --- |
| A | Smokers | Yes | 8 | 10 | √ |
| B | Smokers | Yes | 2 | 10 | √ |
| C | Smokers | Yes | 1 | 30 | √ |
| D | Smokers | Yes | 3 | 70 | √ |

** denotes tissue area engaged by the tumor.
